# Neurological and neuro-ophthalmological manifestations of snake bite: a systematic review

**DOI:** 10.1097/MS9.0000000000001523

**Published:** 2023-11-22

**Authors:** Kamal Pandit, Aastha Rawal, Himang Man Singh Maskey, Gaurav Nepal

**Affiliations:** a Department of Ophthalmology, Maharjgunj Medical Campus, Tribhuvan University, Institute of Medicine, Maharajgunj, Kathmandu; b Department of Pharmacy, Manmohan Memorial Medical College and Teaching Hospital, Kathmandu, Nepal; c Department of Internal Medicine, Maharajgunj Medical Campus, Institute of Medicine, Tribhuvan University, Kathmandu, Nepal

**Keywords:** neurological manifestations, neuro-ophthalmological manifestations, snake bite management, snake bites

## Abstract

**Objective:**

Snakebites, a major health concern in developing countries, affect rural farming communities. Venom, primarily neurotoxin, injected during a snake bite disrupts the nervous system, causing symptoms like muscle weakness, paralysis, altered sensation, and coordination issues. This review focuses on evaluating neurological and neuro-ophthalmological manifestations associated with snakebites.

**Methods:**

A database search was conducted in EMBASE and PubMed for studies published from 2000 to 2023. The investigation centered on examining neurological and neuro-ophthalmological symptoms and signs, treatment approaches, treatment outcomes, and long-term complications of snake bites.

**Results:**

Neurological and neuro-ophthalmological symptoms were common in both neurotoxic and hemotoxic snake bites, especially in neurotoxic cases. Ptosis was a prevalent manifestation across various snake bites, along with respiratory paralysis, limb weakness, dysphasia, and visual disturbances in some instances. However, most patients improved without residual neurological symptoms after treatment.

**Conclusions:**

Understanding patterns of neurological manifestations contributes valuable insights for the comprehensive management of snakebite.

## Introduction

Highlights

- Snake bites are major health concerns in developing countries, mainly rural farming communities.
- Neurological and neuro-ophthalmological manifestations are prevalent regardless of toxin type.
- Physicians should remain vigilant in monitoring and identifying neurological manifestations.
- Neurological and neuro-ophthalmological manifestations improve following the administration of ASV treatment.

Snake bite is a major health concern, particularly in developing countries, affecting mainly rural farming communities^1^. It remains a neglected tropical disease that causes significant morbidity and mortality. Worldwide, an estimated 5.4 million snake bites occur annually, with half being envenoming, leading up to 138 000 preventable deaths and over 400 000 preventable amputations and other permanent disabilities^1^. In the United States (US), poison centres recorded over 7000–8000 snake envenomation in 2019, while in India, an average of 250 000 snake bites are recorded in a single year^2,3^. Snakes are found in deserts, forests, marshes, swamps, lakes, streams, and rivers of difficult terrains. The families of venomous snakes are *Atractaspididae, Elapidae, Hydrophidae*, and *Viperidae* ^3^. Snake venom can be classified into hematotoxic, neurotoxic, necrotoxic, cardiotoxic, and nephrotoxic, depending on the predominant effects of the venom^4^. The venom consists of various components, including protein, enzymes, neurotoxins, coagulants, anti-coagulants, and substances with cytotoxic effects^5^. Venoms are a complex mixture of enzymatic and toxic proteins, which trigger different clinical manifestations depending on the pathophysiological changes of certain species.

Hemotoxins affects the cardiovascular system causing bleeding or coagulation disorder whereas neurotoxins affect the nervous system. When neurotoxin enters the body by a snake bite, it causes neurological symptoms like muscle weakness, paralysis, altered sensation, and coordination issues^4^. It can also impact the visual system, resulting in neuro-ophthalmological manifestations such as blurred vision, double vision, drooping eyelids, and vision loss.

This systematic review aims to outline the nature and severity of neurological and neuro-ophthalmological symptoms and signs, as well as their treatment approaches, outcomes, and long-term complications associated with snake bites.

## Methodology

This review article has been reported in line with PRISMA (Preferred Reporting Items for Systematic Reviews and Meta-Analyses) guideline^6^ and AMSTAR(Assessing the methodological quality of systematic reviews) guideline.

### Study inclusion and exclusion criteria

The following requirements should have been met by the studies: (1) Included patients who had experienced a snake bite (2) Paediatric and adult patients (3) Information on one or more neurological manifestations.

The exclusion criteria were as follows: (1) Animal or in vitro studies (2) Insufficient data and language other than english (3) Duplicate publications (4) Reviews or meta-analyses.

### Search methods and study selection

PubMed and EMBASE databases were searched for studies published from 2000 to 2023. Boolean logic was used for conducting a database search, and Boolean search operators “AND” and “OR” were used to link search terms: “snake bite”, “Neurological manifestation”.

The detailed PubMed search strategy was as follows: (((“neurologic manifestations”[MeSH Terms] OR (“neurologic”[All Fields] AND “manifestations”[All Fields]) OR “neurologic manifestations”[All Fields] OR (“neurological”[All Fields] AND “manifestation”[All Fields]) OR “neurological manifestation”[All Fields]) AND (“snake bites”[MeSH Terms] OR (“snake”[All Fields] AND “bites”[All Fields]) OR “snake bites”[All Fields] OR (“snake”[All Fields] AND “bite”[All Fields]) OR “snake bite”[All Fields]) AND (“loattrfull text”[Filter] AND 2000:2023 [Date - Publication])) NOT ((“case reports”[Publication Type] OR “case report”[All Fields]) AND “loattrfull text”[Filter])) AND (fft[Filter]). There was no restriction on language of publication.

We also searched the reference list of each included study to identify other potential material of interest. All shortlisted studies were then imported to the Mendeley, and duplicates were removed appropriately. Papers were initially reviewed by title, keywords, and abstract by two reviewers (K.P. and H.M.S.M.) independently and subsequently verified with a third reviewer (GN). Articles after the initial screen were subsequently reviewed in full by two reviewers (K.P. and H.M.S.M.). We resolved the final study selection differences between the two primary reviewers (K.P. and H.M.S.M.) by the discussion with a third reviewer (GN). An overall evaluation for potential overlap of the population was conducted based on authorship, hospital setting, and recruitment period. In cases of overlap, studies of higher quality or larger sample sizes were included. The work has been reported in line with AMSTAR (Assessing the methodological quality of systematic reviews) guidelines.

### Data extraction

Two independent authors (K.P. and H.M.S.M.) rigorously reviewed selected studies for systematic review which met our inclusion criteria and extracted the precise information on different headings under one table depicting Author/Published year, study site, study design, sample size, participants (adults, paediatric), age (in years), sex, snake and venom type, neurological and neuro-ophthalmological manifestation, treatment, outcome and late complications were recorded in Microsoft Excel 2013 (Microsoft Corp).

## Results

### Search results and study selection

**Figure 1:**
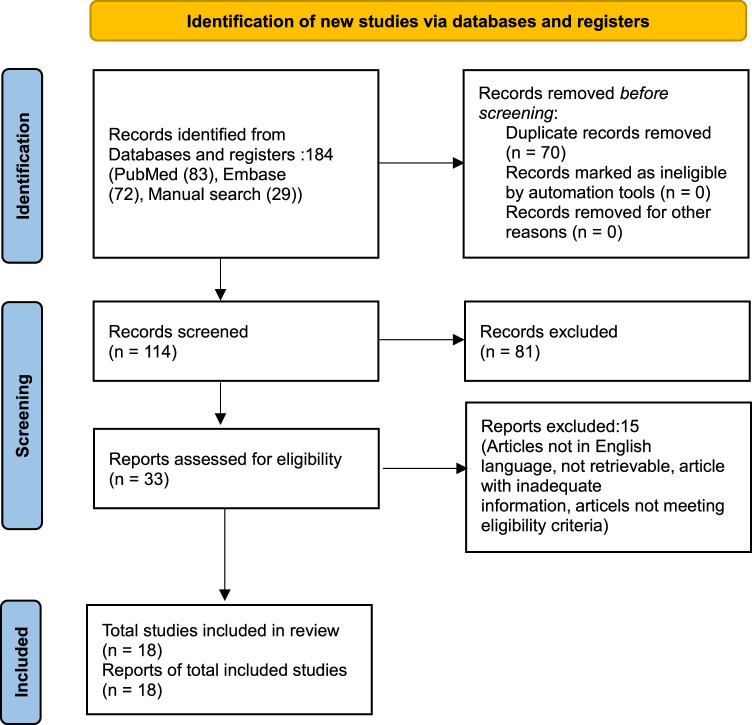
The PRISMA diagram detailing the identification and selection process.

We found 184 studies through electronic database searches (83 from PubMed, 72 from EMBASE) and identified 29 additional studies through manual searches of reference lists and related systematic reviews. After removing duplicates, we screened 114 articles based on titles and abstracts. Subsequently, 33 full-text articles were retrieved and evaluated against predefined inclusion criteria. Ultimately, 18 articles met the criteria and were included in the review. The PRISMA diagram, illustrating the identification and selection process, is provided in Figure 1.

### Study characterizations and patient’s demography

The review encompassed a total of 1,424 patients across 18 studies, comprising 15 observational studies and three case series. The mean age of participants ranged from 6 months to 80 years. Among these studies, four exclusively involved pediatric patients, two exclusively involved adult patients, and 12 included both pediatric and adult participants. The research was conducted in various countries, with five studies from India, four from Sri Lanka, three from the United States, two from Italy, and one each from Thailand, South Africa, Croatia, and Brazil. Table 1 provides a summary of the study characteristics.

**Table 1:** Key methodological characteristics of studies included in this systematic review.

Author and year	Country	Study design	Sample size	Snake type	Neurological manifestations	Treatment	outcomes	Late complications
Marano *et al*. (2021)^6^	Italy	Observational	24 Paediatric (1.5–16.2 years)	Viper	Ptosis 2 (8.3%) Dysarthria 1 (4.2%) Nystagmus 1 (4.2%)	Anti-venom in 12 (55%) of total cases	Improved	None
Tongpoo *et al*. (2018)^7^	Thialand	Observational	78 (1–76 years)	Krait	69 (88.5%) developed neurological signs of motor weakness	Anti-venom:67 (85.90%) of total cases 75.6% of total cases required intubation and ventilator support	Death:5 (7.2%)	None
Hernandez (2019)^8^	USA	Observational	72 children (6 months–15 years)	Types of snake was not indentified	5 (7%) developed progressive weakness	Anti-venom:1 (20%) of those who developed progressive weakness Fasciotomy 2 (40%)	Improved	None
Samprathi *et al*. (2020)^9^	India	Observational	111 Children (5–10 years)	Krait	76 had neuroparalysis (68%) and 51 had EMNS of which 43 (84.3%) had bulbar palsy ,40 (78.4%) had ptosis. 35 had non EMNS of which 16 (45.7%) had bulbar palsy,24(68.5%) had ptosis.	Anti-venom:111 (100%)	With EMNS 7(13.7%) death With non EMNS 3(8.57%) death	None
Seneviratne *et al*. (2002)^10^	Sri Lanka	Observational	56 (13–65 years)	Viper (27) Krait (19) Cobra (3) Unidentified (7)	Ptosis in 48 (85.7%) ophthalmoplegia 42 (75%) limb weakness 15 (26.8%) respiratory failure 10 (17.9%) palatal weakness 6(10.7%) neck muscle weakness 4 (7.1%), delayed sensory neuropathy 1 (1.8%).	Anti-venom 10 cases required mechanical ventilation	Improved 1 (1.79%) death due to Intracerebral haemorrhage	None
Silva *et al*. (2016)^11^	Sri Lanka	Observational	33 (16–78 years)	Krait	Neurotoxicity None—8 (24.24%) Mild—8 (24.24%) Severe—17 (51.52%) Ptosis Partial – 12 (36.36%) Complete—13 (39.4%) Ophthalmoplegia Partial—4 (12.12%) Complete—14 (42.42%) Strabismus—11(33.33%) Facial Weakness—18(54.54%) Neck Flexon Weakness—15(45.45%) Difficulty swallowing—17 (51.51%) Low pitched voice—12 (36.36%) Tidal volume <250 ml—17 (51.51%) Reduced upper limb power—12 (36.36%) Reduced lower limb power (power <5)—5 (15.15%) Diminished or absent deep tendon reflexes 9 (27.27%) Autonomic features—6 (18.18%)	Anti-venom—23 (69.70%) Intubation and mechanical ventilation—17 (51.51%)	Improved Anaphylaxis—6 (18.18%) Intubated for anti-venom reaction—3 (9.10%)	None
Walt (2019)^12^	South Africa	Observational	14 (1–65 years)	Berg Adder	Ptosis, mydriasis and visual disturbances and diminished motor power—13/13(100%) of patients who developed systemic sequelae following envenoming Dysphagia—9/10 (90%) of assessed patients Tendon reflexes diminished—7/9 (77.8%) of assessed patients‘ Respiratory Failure—10/13 (76.9%) of patients who developed systemic sequelae following envenoming	Symptomatic	Improved	None
Karabuva *et al*. (2016)^13^	Croatia	Observational	160 children and adolescent (1--18 y)	Not mentioned	Ptosis—18 (11.2%) 7 (4.4%) ophthalmoplegia, 3 (2.0%) dysphagia, 1 (0.6%) dysphagia and dysphonia	Anti-venom—100% Antibiotics—96% Corticosteriods – 84% Antihistamines—71%	Improve 1 (0.625%)died Fasciotomy—12 (7.5%)	None
Bisneto (2020)^14^	Brazil	Case series	7 (14–49 years)	Coral	dyspnoea/shallow breath (4 (51.14%)), palpebral ptosis (4 (57.14%)), blurred vision (3 (42.85%)), dysarthria (3 (42.85%)) and difficulty to walk (3 (42.85%))	Anti-venom—7 (100%)	Improved	None
Bawaskar *et al*. (2002)^15^	India	Observational	91 (10–62 years)	Kraits—20 (21.97%) Echis—9 (9.9%) Rest unknown 62 (68.13%)	Paralysis—26 (28.6%)	Anti-venom	Deaths—10 (11%)	None
Lonati *et al*. (2014)^16^	Italy	Case series	24 (3–75 years)	Viper	Accommodation troubles and diplopia 24 (100%), ptosis 22 (91.7%), ophtalmoplegia 14(58.3%), dysphagia 5 (20.8%), drowsiness 4 (16.6%), cranial muscle weakness 3 (12.5%), dyspnoea 10 (4.2%)	Anti-venom—19 (79.2%)	Improved	None
Anil *et al*. (2010)^17^	India	Observational	72 (15–65 years)	Krait	Altered sensorium- -4 (5.8%) Ptosis - 70 (97.1%) Weakness of neck flexors—50 (70.0%) Respiratory—10 (10.4%)	Anti-venom NeostigminAtropine	Improved Deaths—2 (2.78%)	None
Roth (2016)^18^	USA	Observational	87 (2–79 years)	Copperheads	Pain, swelling, and disability, weakness, or residual venom effects of involved body parts in all patients.	Anti-venom—57 (65.51%)	Improved Anaphylaxis—2 (2.30%) Urticarial reaction—1 (1.15%)	None
Bawaskar *et al*. (2014)^19^	India	Cross-sectional	141 Age range not mentioned All adults	Krait	37 (26%) victims had neuroparalysis (bulbar palsy). 16 (11.34%)victims had respiratory paralysis and needed ventilator support but seven of these victims died	Anti-venom	12.5% ( 7) died on the way to hospital 13.47% died during treatment	None
Kularatne *et al*. (2014)^20^	Sri Lanka	Observational	55 (20–50 years)	Viper	Proven/Probable grp Ptosis: 30 (55%)/ 109 (71%) Ophthalmoplegia: 30 (55%)/ 111 (72%) Neck Muscle Weakness: 14 (25%)/50 (32%) Respiratory paralysis : 0/ 2 (1%)	Anti-venom Proven Viper Bite : 64% Suspected Viper Bite : 75%	Improved Reaction to Anti-venom: Proven/Probable grp Moderate : 31% / 6% Severe : 14% / 5%	None
Sharma *et al*. (2005)^21^	India	Observational	142 (12–80 years) Paediatric and Adults	Elapid(86 (60.56%)) and Viper (52 (36.61%)) Rest not mentioned in study.	Ptosis : 75 (52.81%) Respiratory involvement: 65 (45.77%) Bulbar weakness: 59 (41.54%) Ophthalmoplegia: 42 (29.57%) LoC : 12 (8.45%)	Equine polyvalent Anti-venom : 119 (83.8%)	Mortality in 5 patients (3.5%) 17 (11.97%) patients had ADR to anti-venom: Anaphylaxis: 2 (1.40%) Pyrexia; 6 (4.22%) Utricaria: 9 (6.33%)	
Kularatne (2002)^22^	Sri Lanka	Observational	210 (10–30) years Paediatric and adults	Krait	Ophthalmoplegia, Ptosis Muscle weakness in 101 (48.1%)patients Paralytic ileus in 42 (20%)patients	Polyvalent Haffkine Anti-venom 101(48.1%) patients required mechanical ventilation	Overall mortality of 7.6% ARDS in 6 (2.85%) patients Arrythmia in 20 (9.52%) patients	None
Vohra *et al*. (2008)^23^	USA	Case Series	47 (5–70) years 7-paediatrics 40-Adults	Rattle Snake	All patients presented with fasciculation in various parts (Face,eyelids,peri oral,oral,lingual,tongue,arms,legs,calf,shoulder,torso) 3(6.38%) Intubated for signs of respiratory distress	Anti-venom: 46 (97.87%) Not Recorded: 1 (2.12%)	Improved	None

ADR, Adverse drug reaction; ARDS, Acute respiratory distress syndrome; EMNS, Early Morning Neuroparalytic Syndorme.

### Snake / Venom types

**Figure 2:**
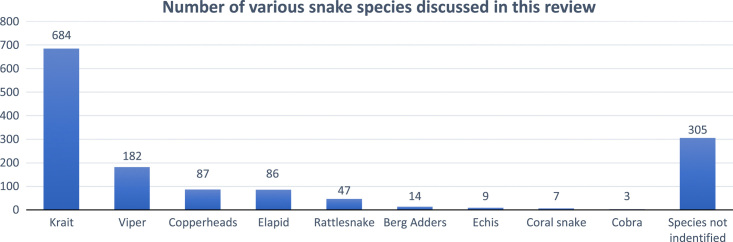
showing number of various snake species discussed in this review.

Out of 1424 cases, 1119 cases specified the snake species: 684 cases were caused by kraits *(Bungarus caeruleus)* (neurotoxin), 182 by vipers *(Viperidae)* (hematotoxin), 87 by copperheads *(Agkistrodon contortrix)* (hematotoxin), 86 by elapids *(Elapidae)* (neurotoxin), 47 by rattlesnakes *(Crotalus cerastes)* (neurotoxin and hematotoxin), 14 by berg adders *(Bitis atropos)* (neurotoxin), 9 by echis *(Echis carinatus)* (hematotoxin), 7 by coral snakes *(Aspidelaps lubricus)* (neurotoxin), and 3 by cobras *(Naja naja)* (neurotoxin). The remaining 305 cases did not identify the snake species. This data is illustrated in the Figure 2.

### Neurological and neuro-ophthalmological manifestations

**Figure 3:**
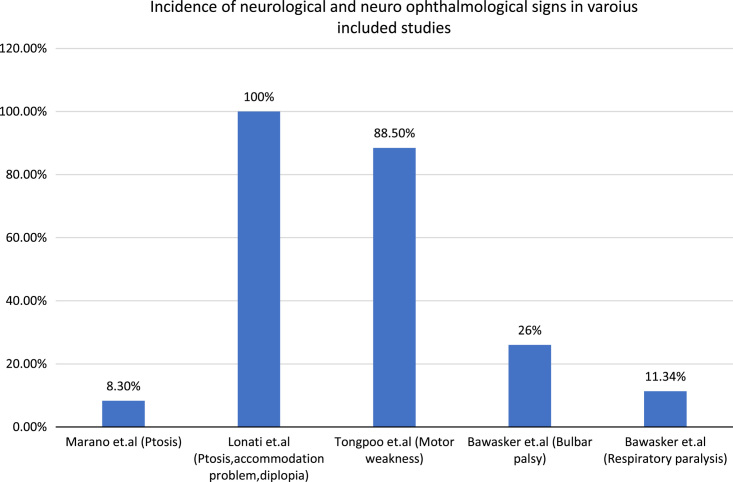
showing incidence of various neurological and neuro-ophthalmological manifestations.

Marano *et al*.^16^ examined 24 cases of paediatric viper bites and found neurological signs in 8.3% of cases, with ptosis being the most common manifestation. Tongpoo *et al*.^7^ examined 78 cases of both paediatric and adult krait bites and found motor weakness to be present in 88.5% of cases. Lonati *et al*.^11^ studied 24 cases of both paediatric and adult viper bites and observed that all patients had accommodation problems and diplopia, with ptosis being the most common neurological manifestation. Similarly, Silva *et al*.^17^ studied 33 cases of krait bites and observed that ptosis was the most common manifestation. Anil *et al*.^19^ examined 72 cases of krait bites and found that most of the cases had ptosis. Bawaskar *et al*.^12^ studied 141 cases of krait bites and observed that 26% of cases had bulbar palsy and 11.34% had respiratory paralysis requiring mechanical ventilation. Additionally, this review included studies on Berg Adder bites, Copperheads bites, rattlesnake bites, and coral bites^13,14,18,23^. The detail can be found on Table 1. Incidence of various manifestations has been shown in Figure 3.

### Treatment

Anti-snake venom (ASV) was administered in all the studies, and the patients were also provided with mechanical ventilation when necessary, except for a study conducted by Walt *et al.* ^18^, who managed 14 cases of Berg Adder bite symptomatically. In the study conducted by Karabuva *et al*.^21^, along with anti-snake venom, antibiotics, corticosteroids, and antihistamines were administered to the patients. However, Anil *et al*.^19^’s study on the effect of neostigmine in krait snake bites showed that the treatment was ineffective.

### Outcome

We found that ASV effectively improved the neurological and neuro-ophthalmological manifestations. However, there have been reports of anaphylaxis caused by ASV in studies conducted by Sharma *et al.* ^20^, Kularatne *et al.* ^10^, Roth *et al.* ^23^, and Silva *et al.* ^17^. The studies have reported mortality rates ranging from 2.48 to 13.7%. Seneviratne *et al*.^24^ reported a case of mortality caused by intracranial haemorrhage. Nevertheless, the majority of patients improved without any residual neurological symptoms.

## Discussion

**Figure 4:**
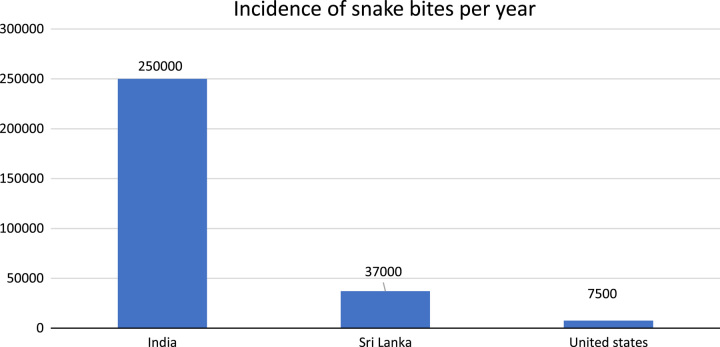
showing the incidence rate of snake bite per year.

Our review showed that snake bites are prevalent in both children and adults. Most of the cases studied were of Krait bite. The majority of cases were reported from India, followed by Sri Lanka and the United States. In India alone, an average of 250 000 snake bites are reported each year^3^, while Sri Lankan hospitals admit around 37 000 snake bite cases annually^25^. This has been shown in Figure 4.

Neurotoxins present in snake venom bind to neuromuscular junctions both pre-synaptically and post-synaptically, resulting in muscle weakness. For instance, alpha-bungarotoxin found in krait venom binds to acetylcholine receptors, leading to a reduction in acetylcholine receptor sites in the postsynaptic membrane^26^. On the other hand, alpha-cobra toxin produces features of myasthenia gravis in experimental animals due to its similar action^27^. Neurotoxicity is caused by the pre-synaptic actions of phospholipase A2 enzyme and Beta-bungarotoxin^22,28^.

Hematotoxic venoms, on the other hand, can cause neurological symptoms like ptosis, ophthalmoplegia, and weakness due to three mechanisms: presynaptic inhibition of neurotransmission by phospholipase A2 in Russell’s viper venom, intracranial haemorrhage, or acute brain infarction^29,30^.

Most patients in this review report ptosis followed by neurological weakness of limbs and ophthalmoplegia, while nystagmus, delayed neuropathy, and dysarthria were reported in fewer patients. A moderate number of patients reported decrease in the visual acuity with snake bites. In neurotoxic snake bites, ptosis followed by neuroparalysis are the most common ocular signs and symptoms^19,31^, while hematotoxin snake bites can cause symptoms ranging from accommodation deficiency and ptosis to ophtalmoplegia, neck muscle weakness, and dysphagia^10,20^. According to Silva *et al*.^17^, patients who do not develop bulbar weakness or respiratory paralysis within 12 hours of the bite are unlikely to experience severe paralysis.

Several authors have reported muscle weakness after snake bites, which clinical and electrophysiological studies have attributed to defective neuromuscular transmission^32,33^. However, there have also been cases where muscular weakness was caused by rhabdomyolysis^34^. Snake bites can lead to potentially fatal respiratory muscle weakness, with krait bites being particularly notorious for rapidly causing respiratory failure^24^. However, in a series of ten patients with respiratory failure, all were successfully managed with mechanical ventilation, underscoring the importance of timely intervention^24^. Snake bites can also lead to Guillain-Barre syndrome and delayed neuropathy^31^. Neurotoxin snake bites commonly cause high blood pressure and tachycardia, which may be due to decreased parasympathetic activity^35^. Electrolyte imbalances, including hyponatremia, are also recognized clinical manifestations^36^.

ASV was administered to patients with systemic features, and our review revealed that it had a positive effect. Nevertheless, instances of anaphylaxis to ASV were also noted and were handled appropriately. The use of anti-venom has a high incidence of anaphylaxis^37,38^, as demonstrated in this review, which may limit its efficacy when widely used for snake bites and envenomation. The administration of anti-venom must be done in a high-care environment with constant monitoring and should be preceded by the administration of intramuscular adrenaline^39^.

The syndromic approach to managing snake bites, as popularized by Blaylock in 2005, divides management into three clinical syndromes that correspond to the three venom types^40,41^. The therapeutic triad, which includes elevation, intravenous fluids, and analgesia, is the mainstay of conservative management when the type of snake involved is unknown^40^. In severe cases of progressive painful swelling or bleeding, anti-venom administration and fasciotomy may be necessary^42^. Low dose anti-venom has been found to be as effective as high dose anti-venom for both neurotoxic and hematotoxic snake bites^43^. Early, empirical anti-venom is lifesaving, but good supportive care with mechanical ventilation is equally important and can lead to a good outcome^44^. The use of anti-coagulants in viper bites is controversial, with some reports suggesting it may increase the risk of haematoma and functional impairment^9,45^. The role of anticholinesterases in reversing neuroparalysis is also debated, with some studies showing ineffectiveness^8,31^.

Most cases in the reviewed studies recovered without neurological deficits. However, mortality rates were higher with neurotoxic snake bites compared to hemotoxic ones. Factors like younger age, ptosis, cardiac arrest, and lack of PICU beds were associated with higher mortality rates in children with EMNS, but prompt recognition, respiratory support, and PICU care can decrease mortality^15^.

To effectively prevent and treat snake bites, it is important to establish a proper documentation system such as a Snake Bite Registry that includes information on snake species, patient history, clinical signs, treatment, and final outcome. The North American Snakebite Registry (NASBR) is an example of such a registry^46^.

## Conclusion

Ptosis is a prevalent neurological symptom in various snakebites, often accompanied by respiratory paralysis, limb weakness, dysphasia, and visual disturbances in certain cases. Notably, improvements are observed after ASV treatment, with almost no residual manifestations.

## Ethical approval

Not Applicable for Systematic Review.

## Consent

Informed consent was not required for this systematic review.

## Source of funding

There is no any source of funding for this study.

## Author contribution

K.P.: conceptualization, data curation, formal analysis, methodology, resources, validation, visualization, writing—original draft. A.R.: formal analysis, validation, methodology, writing—review and editing. H.M.S.M. and G.N.: conceptualization, resources and validation.

## Conflicts of interest disclosure

None.

## Research registration unique identifying number (UIN)

Name of the registry: Research registry Hyperlink https://www.researchregistry.com/browse-the-registry#home/registrationdetails/64c2a232f693a200273b1011/.

UIN: researchregistry9326.

## Guarantor

Kamal Pandit.

## Provenance and peer review

Not commissioned, externally peer-reviewed.

## Data availability

The authors declare no conflict of interest.
